# Neurodevelopmental outcome and respiratory management of congenital central hypoventilation syndrome: a retrospective study

**DOI:** 10.1186/s12887-020-02239-x

**Published:** 2020-07-13

**Authors:** Tomomi Ogata, Kazuhiro Muramatsu, Kaori Miyana, Hiroshi Ozawa, Motoki Iwasaki, Hirokazu Arakawa

**Affiliations:** 1 Department of Pediatrics, Graduate School of Medicine, Gunma University, 3-39-15 Showa-machi, Maebashi City, Gunma 371-8511 Japan; 2 Department of Pediatrics, Jichi Medical University, Tochigi, Japan; 3 Department of Pediatrics, Japanese Red Cross Medical Center, Tokyo, Japan; 4 Shimada Ryoiku Center Hachioji, Tokyo, Japan; 5 Division of Epidemiology, Center for Public Health Sciences, National Cancer Center, Tokyo, Japan

**Keywords:** Apnea, Infant, Tracheostomy, Intellectual development, Bilevel continuous positive airway pressure, *PHOX2B*

## Abstract

**Background:**

Congenital central hypoventilation syndrome (CCHS) is a rare disease characterized by sleep apnea. Anoxia often occurs soon after birth, and it is important to prevent anoxia-mediated central nervous system complications; however, data on the relationship between respiratory management and the prognosis for intellectual development of patients with CCHS is not well yet investigate.

**Methods:**

We performed a retrospective chart review cohort study of patients with CCHS in Japan. We investigated the risk and prognostic factors for developmental outcomes and examined the disease in terms of its symptoms, diagnosis, complications, and treatment.

**Results:**

Of the 123 patients with CCHS included in the survey, 88 patients were 6 years old and older. They were divided into two group based on their intelligence quotient. Those treated using positive-pressure ventilation via tracheostomy in the first three months of life had a better developmental prognosis than those managed via tracheostomy after three months of age and those treated by ventilation using mask (OR = 3.80; 95% CI: 1.00–14.37, OR = 4.65; 95% CI: 1.11–19.37). There was no significant difference in physical development (*P* = 0.64).

**Conclusions:**

The best respiratory treatment for patients with CCHS is ventilation via tracheostomy, initiated ideally before the age of three months.

## Background

Congenital central hypoventilation syndrome (CCHS) is a rare neurocristopathy characterized by sleep apnea and an autonomic nervous system dysfunction in the neonatal period; it was first reported by Mellins et al. in 1970 [1]. The estimated incidence of CCHS is approximately 1 in 200,000 live births [2]. It is related to mutations in the paired-like homeobox 2B (*PHOX2B*) gene [3, 4] and is associated with the Hirschsprung disease, neuroblastoma, and autonomic nerve dysfunctions [5]. The amount of data currently available on the relationship between intellectual development and disease state in CCHS patients is limited.

In the European Union and North America, respiratory ventilation of CCHS is managed by using positive-pressure ventilators via tracheostomy. Other forms of ventilation management for patients with CCHS include bilevel continuous positive airway pressure (BiPAP), negative-pressure ventilators, and diaphragm pacing. A policy statement by the American Thoracic Society recommends positive-pressure ventilation via tracheostomy over several years, beginning during the first days of life [6]. However, there have been recent reports of CCHS patients treated after birth with BiPAP [7, 8]. In Japan, there are no guidelines for CCHS respiratory management, and many children are currently managed with BiPAP.

In 2014, we reported on the intellectual development of 23 CCHS patients and found that treatment played an important role in the prevention of intellectual disability caused by hypoxemia and hypercarbia after birth [9]. In the present study, we conducted a survey of CCHS patients in Japan to assess the range of medical care being received and the developmental abilities of the patients in order to determine the factors that are most strongly associated with an intellectual development.

## Methods

Between November 2013 and July 2014, the initial questionnaires were mailed to all 519 certified training hospitals (3 to 5 pediatricians each) of the Japan Pediatric Society. In addition, identical questionnaires were mailed to 154 hospitals in which the pediatric neurologists worked. Between July 2014 and October 2015, detailed questionnaires that aimed to assess symptoms, diagnosis, complications, medical treatment, and developmental outcome in CCHS patients were mailed to 174 physicians (in 134 hospitals) who had previously responded that they had prior experience or current experience of medical care for these patients. The questionnaire elements included the patients’ age at the time of onset of symptoms, age at the time of diagnosis, methods used in the diagnosis, family history, medical condition, types of ventilation used, types of school attended, and physical and intellectual development. In Japan, the diagnosis of CCHS patients, whose primary complaint was sleep apnea and who were referred from regional general pediatricians, was confirmed genetically and clinically, except for those in which neural, muscular, and cardiovascular diseases were concomitant with severe apnea.

Patients aged six years and older were divided into two groups on the basis of prognosis of their intellectual development. Those who attended (or had previously attended) regular classes school or who had an intelligence quotient (IQ) ≥ 75 were assigned to the no intellectual disability group, whereas those who attended a special education class or whose IQ was < 75 were assigned to the intellectual disability group.

In the group comparisons between the patients managed with tracheostomy and those managed with non-invasive ventilation, the Mann–Whitney *U*-tests were used to compare the age at the onset of symptoms and the age at CCHS diagnosis. Analysis of variance (ANOVA) was used to assess the differences in the mean ages of the CCHS patient groups in the study, while the Chi-squared (χ^2^) tests were used for other factors. Continuous variables have been presented as means ± standard deviation (SD). Multivariate logistic regression analysis was used to examine the mental development outcomes in relation to the respiratory management of the CCHS patients, i.e., via tracheostomy (before three months in life or after three months in life) or with a non-invasive ventilation. Odds ratio (OR) and 95% confidence interval (CI) were adjusted for potential confounding factors, namely the age of the patients and daytime hypoventilation. We used the SPSS version 23.0 (IBM) for all analyses. All reported *p*-values were 2-sided, and the significance level was set at *P* < 0.05.

## Results

### Demographic characteristics

**Fig. 1:**
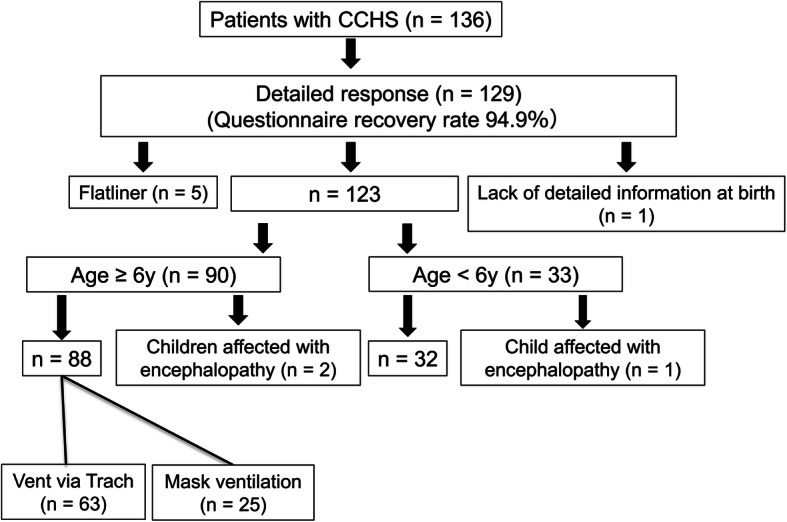
Flowchart representing the questionnaire-based enrolment of subjects into the study and the study subgroups. Numbers of patients > 6 years of age represents CCHS children followed up after the survey

The response rate to the first questionnaire was 95%. We established that there were 136 CCHS patients in Japan. Detailed questionnaire responses were received from the physicians of 129 of these CCHS patients, corresponding to a response rate of 95%. Five of these patients had died and we were unable to obtain sufficient data for one patient; the other 123 patients were treated in Japan (Fig. 1). There were six fraternal cases and seven familial cases. The gender ratio (male/female) in these CCHS patients was 71 / 52. The mean age of each was 12.3 ± 8.1 years and 11.4 ± 8.3 years, respectively. All the relevant data pertaining to patients have been addressed in the analysis.

### Ventilation methods

The distribution of the patients’ ages and the respiratory care methods used at the time of the first questionnaire survey were as follows: 92 patients were managed with ventilation via tracheostomy and 31 with BiPAP ventilation using a mask. There was no difference between the two respiratory management groups in terms of age (12.3 years ±9.05 years vs 8.94 years ±4.93 years, respectively, *P* = 0.051) or sex (male: female ratio = 56:36 vs 15:16; *P* = 0.15). Thirteen patients required a mechanical ventilation support either during sleep or 24 h/day. Of the 92 patients treated using a positive-pressure ventilation via tracheostomy, 34 (37%) patients used a speech cannula. Many patients were able to talk using air leaked to the upper respiratory tract. Nineteen patients switched from respiratory treatment with tracheostomy to a non-invasive positive-pressure ventilation; the age range for this switch was 4 years–25 years, with the mean age being 10.4 years ±5.8 years. The treatment of one patient was changed from ventilation via tracheostomy to diaphragm pacing while sleeping. Of the 31 patients who had never been tracheostomized, 6 were treated with BiPAP via full face mask, 14 used a nose–mouth mask, and 11 used a nose mask.

### Diagnosis

The methods used for the diagnosis of CCHS in the 123 patients have been shown in Table 1.

**Table 1:** Methods used in the diagnosis of CCHS (*n* = 123)

	Number	Percent
Clinical manifestations	102	82.9
SpO_2_ monitoring	82	66.7
*PHOX2B* gene mutation test	74	60.2
Blood gas analysis	62	50.4
Ventilatory response to CO_2_	24	19.5
End-tidal CO_2_	16	13.0
Tidal volume monitoring	9	7.3
Percutaneous CO_2_ monitoring	2	1.6
Clinical manifestations + SpO_2_ monitoring	71	57.7
Clinical manifestations + Blood gas analysis	59	48.0
Clinical manifestations + Ventilatory response to CO_2_	20	16.2

*PHOX2B* gene mutations were detected in 71 (96%) of the 74 patients who underwent a genetic test. Other patients were diagnosed with CCHS by a composite approach, such as clinical manifestations, SpO_2_ monitoring during sleep, blood gas analysis, the CO_2_ ventilation response, end-tidal CO_2_, ventilation volume, and percutaneous CO_2_ monitoring. Table 2 depicts the distribution of age at the times of onset of symptoms and age at diagnosis.

**Table 2:** Age at the onset of symptoms and age at diagnosis

	With tracheostomy		Without tracheostomy	
Age at the onset of symptoms	*n*	%	*n*	%
Day 0	68	74.7	19	61.3
Day 1–5	12	13.2	3	9.7
Before 1 month	8	8.8	3	9.7
After 1 month	3	3.3	6	19.4
Total	91		31	
Age at diagnosis				
Before 2 months	61	76.3	15	57.7
3–5 months	12	15.0	5	19.2
6–11 months	3	3.8	1	3.8
After 1 year	4	5.0	5	19.2
Total	80		26	

In terms of the age at the time of onset of symptoms, there were no significant differences observed between the two groups of patients, i.e., patients with tracheostomy and patients with mask ventilation (*P* = 0.07). However, in terms of the age at diagnosis, patients with tracheostomy were diagnosed earlier than patients with mask ventilation (*P* = 0.04). There is one patient whose age at the onset of symptom is unknown. Most patients (97%) experienced the onset of symptoms within one month after birth, and 72% of the patients received a diagnosis within three months of birth.

### Complications

Table 3 shows the range of complications experienced by the patients with CCHS. The Hirschsprung disease was present in 53 patients (43%). Epilepsy was present in 23 patients (18.7%). Thirty-two patients with CCHS had an autonomic nervous system disorder, such as arrhythmia and breath-holding spells, excessive sweating, cyanosis when concentrating and defecating, and abnormal regulation of body temperature. Other complications included tracheomalacia, pulmonary hypertension, gastroesophageal reflux, constipation, atrial/ventricular septal defect, strabismus, and midfacial hypoplasia. Although neuroblastoma is a known complication of CCHS, there were no cases of neuroblastoma in this study.

**Table 3:** The range of complications experienced and percentage of the affected individuals in 123 CCHS patients

Daytime hypoventilation	Number	Percent^a^
	31	25.2
Tracheomalacia	13	10.6
Pulmonary hypertension	7	5.7
Hirschsprung disease	53	43.1
Gastroesophageal reflux disease	5	4.1
Constipation	15	12.2
Arrhythmia	24	19.5
ASD/VSD	2	1.6
Epilepsy	23	18.7
Autism	15	12.2
Learning disorder	5	4.1
AD/HD	3	2.4
Speech development delay	14	11.4
Encephalitis encephalopathy	3	2.4
Autonomic nervous system disorders	7	5.7
Breath-holding spell	8	6.5
Strabismus	16	13.0
Midfacial hypoplasia	12	9.8

^a^Many patients had certain complications. The percentage is the ratio of the number of patients with complications to all patients

### Physical and intellectual development

Of the 123 children and young adults with CCHS, we examined the physical and the intellectual development of the 90 patients aged six years and older, including the children who were followed up. However, two of these patients were excluded because they had experienced encephalitis and encephalopathy before the school-going age (Fig. 1).

In 88 patients aged six years and older with CCHS, 23 subjects described in 2014 were included [9]. There were 63 patients with tracheostomy and 25 treated using BiPAP. Among the patients who underwent tracheostomy, 92% were in the normal physical development group (*n* = 58); among patients treated using BiPAP, 92% were in the normal physical development (*n* = 23). There was no significant difference in terms of physical development between patients treated with ventilation via tracheostomy and those treated with BiPAP (*P* = 0.64).

Responses on the learning and school survey items indicated that 47% (*n* = 41) of the patients older than 6 years (*n* = 88) were in the no intellectual disability-group (i.e., they attended regular lessons or had an IQ ≥ 75), whereas 53% (*n* = 47) of the patients were in the intellectual disability-group (i.e., they attended a special education class or had an IQ < 75). We analyzed the association between these groups and the respiratory care method the patients received by multivariable analysis. The respiratory care method was divided into three groups: those treated using a positive-pressure ventilation via tracheostomy in the first three months of life, those managed via tracheostomy after three months of life, and those treated using the BiPAP ventilation with a mask. In 35 children were divided by type of IQ test, 14 children measured by Wechsler Preschool and Primary Scare of Intelligence and Wechsler Intelligence Scare for Children, 7 children measured by Binet test, 7 children measured by other tests, 7 children were unknown. Because the age of the patients and daytime hypoventilation differed significantly between the treatment groups (*P* < 0.01, *P* = 0.04), ORs were calculated using them as potential confounding factors. The patients treated using a positive-pressure ventilation via tracheostomy in the first three months of life showed better developmental prognoses than the patients managed via tracheostomy after three months of age (OR = 3.80; 95% CI: 1.00–14.37) or patients treated using the BiPAP ventilation via a mask (OR = 4.65; 95% CI: 1.11–19.37) (Table 4).

**Table 4:** Results of the odds ratio analysis for factors that were possibly associated with an intellectual disability (i.e., IQ < 75 or attendance in special education classes)

	Number of patients	Intellectual disability	No intellectual disability		
	*(n = 88, %)*			Odds	
		*n*	*n*		95% confidence interval
	*Male/Female*			ratio*	
With tracheostomy (before 3 months in life)	16 (18) 9/7	4	12	1.00	(Reference)
With tracheostomy (after 3 months in life)	47 (53) 33/14	29	18	3.80	1.00–14.37
Without tracheostomy	25 (28) 12/13	14	11	4.65	1.11–19.37

^a^Adjusted for patients’ age and hypoventilation on awakening

Confounding factors included the age of the patients and hypoventilation on awakening, which differed significantly between the treatment groups

## Discussion

Our results showed that patients treated using a positive-pressure ventilation via tracheostomy in the first three months of life had a better developmental prognosis than either group of patients managed via tracheostomy after three months of age or treated using ventilation by a mask. In comparison, an epidemiological survey including 196 patients with CCHS from 19 countries had reported that 61.7% of the patients were ventilated via tracheostomy and 14.3% of the patients had never been tracheotomized [6]. Of the patients included in our study, 25.2% had never been tracheotomized. This indicates the remarkable improvement of the performance of ventilation using a mask in the patients’ homes.

CCHS was diagnosed through multiple methods and patients as young as infants and pre-school children underwent genetic testing. Children who experienced respiratory symptoms soon after birth often received an early diagnosis of CCHS. The most common complication was the Hirschsprung disease. In previous studies, the prevalence rates for epilepsy have been reported as 5.1–15.4 per 1000 in the general population [10, 11]. Our study showed that 18.7% of patients with CCHS had epilepsy, which was found to be higher than that in the general population. As such, CCHS may be an important determining factor of epilepsy. All patients with midfacial hypoplasia received a ventilation by mask. Midfacial hypoplasia was associated with a high incidence of patients who started their treatment during infancy with ventilation using a mask. Disfunction of the autonomic nerves resulted in arrhythmia, excessive sweating, the abnormal regulation of body temperatures, and cyanosis when concentrating and defecating. An episode of an autonomic disorder was key to the patients’ interview. Breath-holding causes bradycardia and arrhythmia, in particular when patients with CCHS participate in swimming activities.

Each method of respiratory management has its advantages and disadvantages. Respiratory management via tracheostomy has the advantage of ensuring that there is no aspiration into the airway. During infancy, children frequently experience an upper respiratory tract inflammation. Performing a suction directly is of great importance for certain types of respiratory management via tracheostomy. Another advantage is the short time taken to attach the respiratory ventilation apparatus. During infancy, children fall asleep suddenly and frequently during the day. Coping quickly with these sudden naps reduces the likelihood of hypoventilation events. The disadvantages of this type of respiratory management are the criteria for a surgical procedure, vocal disorder, tracheal granulations, and stenosis. In this study, patients overcame vocal disorders by using speech cannulas and spoke using an air leak to the upper respiratory tract. In addition, the most common causes of tracheostomy-related problems were cannula obstruction and an accidental decannulation. Children who required ventilation via tracheostomy usually required full-time care during the first few years to avoid an airway aspiration.

The advantages of treatment with ventilation using a mask are that it requires no surgical procedure, allowing early discharge, and that caring for the equipment at home costs less than that for tracheostomy [7, 8]. However, sputum and snivel make ventilation more difficult due to an upper respiratory tract inflammation; this is the greatest disadvantage of ventilation using a mask. Because masks have to form a tight seal around the patient’s face and nose, a high incidence of midfacial dysplasia and reversed occlusion have been associated with masks used during infancy [12, 13]. If the degree of adhesion of the mask to the face is low, air can leak out. Some children who use masks are very unwilling to hold the mask on their faces. Recently, there have been reports in the literature about avoiding these disadvantages in ventilation treatment using masks for patients with CCHS. Since there is currently no guideline in Japan regarding the respiratory treatment for CCHS, many doctors choose ventilation treatment using a mask for patients with CCHS [6, 14].

Zelko described deficiencies in the intellectual abilities of 20 school-aged children of CCHS [15]. Charnay found that, on an average, 31 pre-school children with CCHS showed lower intellectual and physical abilities compared to that in the population average [16]. Vanderlaan described 196 patients with CCHS, of whom 45% exhibited developmental delay, 30% had learning disorder, and 13% had attention deficit/hyperactivity disorder [6]. Marcus [17], Oren [18], and Silvestri [19] reported learning disabilities and intellectual disabilities in smaller CCHS samples. In addition, Shimokaze reported that Japanese children with the 25/20 genotype had a high rate (42%) of intellectual disability [20]. Weese–Mayer reported that this result was caused by an inappropriate respiratory management [21]. Although these reports described the association between CCHS and development prognosis, the amount of data on the relationship between the prognosis for intellectual development and the patient’s state of CCHS remains limited.

Our results showed that patients treated using a positive-pressure ventilation via tracheostomy in the first three months of life had a better developmental prognosis than patients managed via tracheostomy after three months of age or patients treated using ventilation by a mask. There are several possible reasons for this. First, extubation and wearing masks may have been attempted more than once for patients with CCHS who underwent a tracheostomy in late infancy. A second possibility is the ability of ventilation via tracheostomy to cope quickly with frequent daytime naps during neonatal life and infancy. Third, as previously described, children with CCHS may likely experience more severe hypoventilation with respiratory tract infections. Airway secretion can consistently be removed when patients are managed by ventilation via tracheostomy.

In general, patients managed by ventilation using a mask have a less severe respiratory indication than the patients managed by ventilation via tracheostomy. Hypoventilation causes intellectual disability, and patients with mild symptoms managed by ventilation using a mask may be expected to experience less delay in their development. However, in this study we demonstrated that respiratory management with ventilation via tracheostomy during early infancy resulted in less intellectual disability. The management with ventilation via tracheostomy was changed to using a mask in some patients with CCHS aged 3 years–6 years [9, 22]. Generally, it is difficult for patients under 7 years of age to make this transition. Patients younger than 7 years may remove the mask themselves because they may feel uncomfortable and do not readily understand the intended treatment [23].

We suggest that patients with CCHS should be managed by ventilation with a tracheostomy during the first three months of their life and that this should be changed to ventilation using a mask or a diaphragm pacer [24] or an external-negative pressure ventilation [25] during school age. This course of treatment has a lower risk of causing a delay in the intellectual development and a lower risk of midfacial hypoplasia. Avoiding an intellectual disability and complications will improve the quality of life of patients with CCHS.

This study has some limitations. It was a retrospective analysis performed on the basis of a nationwide questionnaire survey of almost all CCHS cases in Japan. In 88 patients with CCHS aged six years and older, 35 children were grouped by an IQ test, while 53 children were grouped by special education needs or by regular schooling/classes. To define the incidence and the severity of intellectual disability, we need to have more extensive prospective studies. We will survey IQ of patients with CCHS assessed by Wechsler Intelligence Scare for Children at 7 to 10 years old. Recently, it is reported that some brain deficits include the hippocampus and anterior thalamus damage in CCHS patients were found by magnetic resonance imaging (MRI) [26]. Brain MRI studies are also necessary to examine the prognosis of intellectual development. In addition, our results may not correlate with those of other countries on the grounds of racial differences, as well as different healthcare systems and medical technologies.

## Conclusions

In this comprehensive study of patients with CCHS in Japan, respiratory management with ventilation via tracheostomy during early infancy resulted in a lower degree of intellectual disability. The best respiratory treatment for patients with CCHS is to begin treatment with ventilation via tracheostomy before three months after birth. However, as the patient becomes older, the treatment should be changed to ventilation using a mask, a diaphragm pacemaker, or to an external-negative pressure ventilation.

## Data Availability

The datasets used and/or analyzed during this study are available from the corresponding author on reasonable request.

## Abbreviations

BiPAP: Bilevel continuous positive airway
CCHS: Congenital central hypoventilation syndrome
CI: Confidence interval
IQ: Intelligence quotient
OR: Odds ratio
*PHOX2B*: Paired-like homeobox 2b
